# Supplementary material for: Unveiling novel double-negative prostate cancer subtypes through single-cell RNA sequencing analysis
Source: NPJ Precis Oncol. 2024 Aug 2;8:171. doi: 10.1038/s41698-024-00667-x (PMC11297170; doi:10.1038/s41698-024-00667-x)
Supplement: Supplementary file 1 — Supplementary Information [file 41698_2024_667_MOESM1_ESM.pdf]

Supplementary Note 1

1. Charactering the previously known populations within the HuPSA and MoPSA datasets

The luminal populations were defined by the expression of prostatic luminal epithelial marker genes FOXA1, HOXB13, and NKX3-1 (Figure 1C). The transcriptomes of luminal cells were extracted from HuPSA and MoPSA datasets and visualized on UMAPs for higher resolution (Supplementary Figure 2A & 2B). In HuPSA, the luminal cells consisted of seven populations including normal (contributed by normal, normal-adjacent, benign, and some AdPCa samples), three AdPCa-AR^{+} groups, AdPCa-AR^{high}, AdPCa-AR^{low}, as well as highly proliferating groups. The AdPCa-AR^{+}-1 population only slightly deviated from normal cells on UMAP (Supplementary Figure 2A). Its inferred copy number variation (iCNV) pattern was also similar to that of normal cells (Supplementary Figure 1C). This population may represent low grade PCa, and cells in this population are still at the early stage of transformation. In contrast, the AdPCa-AR^{+}-2 population harbored higher level of iCNVs (Supplementary Figure 1C). The highest iCNVs level was present in the AdPCa-AR^{high} population (Supplementary Figure 1C), suggesting a genome instability in these cells. The AdPCa-AR^{+}-2 and AdPCa-AR^{high} populations were mostly derived from advanced PCa samples including CRPCa, mCRPCa, and CSPCa (Supplementary Figure 2E). Another population that was mostly derived from advanced PCa was AdPCa-AR^{+}-3 (Supplementary Figure 2E). It departed from the main AdPCa cluster (Supplementary Figure 2A), but its iCNV pattern did not significantly differ from that of normal cells (Supplementary Figure 1C), suggesting that genome instability may not be a driving mechanism of cancer progression in these cells. It is interesting to note that the AdPCa-AR^{+}-3 population expressed NPY, a gene widely expressed in the central nervous system, as well as two smooth muscle markers TAGLN and ACTA2 (Supplementary Figures 2C & 2D). This suggests that while still expressing luminal genes such as HOXB13, FOXA1 and NKX3-1, AR^{+}-3 population may have acquired certain mesenchymal features. These cancer cells are likely driven by epigenetic reprogramming.

The AdPCa-AR^{low} population, although low in AR expression, still expressed KLK3 and NKX3-1, two AR target genes, at fair levels (Supplementary Figure 2C), indicating an active AR signaling in these cells.

The proliferating population exhibited high proliferative activity as indicated by the high percentage of cells at predicted G2M/S phases of the cell cycle as well as the high expression of MKI67 and TOP2A (Figure 1C). The cells in this population were mostly derived from CRPCa and mCRPCa as well as CSPCa samples (Supplementary Figure 2E). This population exhibited active AR signaling, indicated by the expression of AR and AR target KLK3 (Supplementary Figure 2C).

The luminal populations in MoPSA included LE/AdPCa -1, AdPCa -2 & -3, and highly proliferating populations, as well as a unique Spink1 population that was not detected in HuPSA (Supplementary Figure 2B). The Spink1 population was predominantly contributed by the prostates of wild-type mice (Supplementary Figure 2E) and it harbored low iCNVs (Supplementary Figure 1D). Because of its absence in HuPSA and the relatively normal molecular profiles, the Spink1 population will not be further characterized in this study. All the other luminal cells in MoPSA expressed Hoxb13 and Foxa1 (Figure 1C), like those in HuPSA. But NKX3-1, a prostate differentiation marker, was only expressed at high levels in the normal LE/AdPCa-1 population (Figure 1C), suggesting a loss of differentiation state in the other populations. In addition, unlike the luminal populations in HuPSA, all of which expressed the AR target genes (such as KLK3) regardless of AR expression status, only LE/AdPCa-1 but not any other luminal populations in MoPSA expressed AR target gene, Pbsn (Supplementary Figure 2C). Given that the LE/AdPCa-1 population predominantly originated from wild-type mice, with only a limited number of

cells originating from genetically engineered mice, and it harbored low iCNVs (Supplementary Figure 2E & Supplementary Figure 1D), this population may represent normal prostate luminal epithelia (LE) and the cells at an early stage of transformation. The other AdPCa populations, including AdPCa -2 & -3 and highly proliferating populations, on the other hand, were predominantly contributed by GEM (Supplementary Figure 2E) and harbored slightly higher level of iCNVs than the LE/AdPCa-1 population (Supplementary Figure 1D).

2. MMP7 and WFDC2 are novel markers for prostate club cells

The prostate club cell population was observed in HuPSA but not in MoPSA. This population, recently identified in the prostate through scRNAseq analysis, was named after airway club cells in the lung, a dominant secretory cell population characterized by the expression of the SCGB1A1 and MMP7[1,2]. In the prostates, similar to the KRT7 population, club cells were clustered together with basal cells on UMAP but did not express basal epithelial markers (Supplementary Figure 3A & Figure 1C). Instead, they expressed markers of airway club cells including MMP7, WFDC2 and SCGB1A1 (Supplementary Figure 3A).

The HuPSA club population exhibited low iCNVs (Supplementary Figure 1C), suggesting that these cells are likely non-cancer cells, consistent with literatures[2]. As illustrated in Supplementary Figure 3A, MMP7 was expressed in the club population, whereas WFDC2 was expressed in both club and basal populations. This expression pattern was confirmed by IHC staining. MMP7 expression was detected in in the apical side of a subset of luminal epithelial cells in human benign prostatic hyperplasia (BPH) samples (Supplementary Figure 3B). On the serial section, WFDC2 expression was detected in the basal cells as well as in the areas positive for MMP7 immunostaining (Supplementary Figure 3B). Moreover, dual immunofluorescence staining results showed that MMP7 was co-expressed with luminal marker KRT8 but not basal marker KRT5 (Supplementary Figure 3C), indicating that the club population, although clustered together with basal cells, are luminal epithelial cells.

3. Mouse NEPCa uniformity vs. human NEPCa subpopulations

NEPCa cells from both HuPSA and MoPSA expressed NEPCa markers INSM1 and NCAM1 (Figure 1C). For a more detailed analysis of NEPCa cells, the transcriptomes of these cells were extracted from the HuPSA and MoPSA datasets, and subsequently visualized on UMAPs (Supplementary Figure 4A). While the mouse NEPCa population was relatively uniform, the human NEPCa cells can be further divided into distinct sub-populations. Through unsupervised clustering and examination of marker genes' expression, the HuPSA NEPCa cells could be further divided into five sub-populations based on the expression of marker genes or the pathways enriched, including CHGA, G2M, TOX, STAT3, and NEUROD1 (Supplementary Figures 4A-4C). It is noteworthy that the expression of marker genes was not mutually exclusive among these groups.

The CHGA subpopulation expressed high levels of the NE marker CHGA (Supplementary Figure 4D). It represents the canonical NEPCa phenotype. The other NEPCa sub-populations expressed relatively low but non-negligible levels of CHGA.

The G2M sub-population was characterized by the high expression of the signature genes associated with cell cycle G2 & M phases such as TOP2A, MKI67, CENPF/W, CKS1B, CKS2 and STMN1. Concurrently, this sub-population displayed heightened transcriptional activity of FOXM1 (Figure 6C), a transcription

factor linked to cell proliferation. In contrast, the other subpopulations exhibited an even distribution of genes related to G1, S and G2M phases. Notably, multiple genes enriched in this sub-population are involved in DNA damage repair (DDR) pathway, including ASPM, CDKN2A/D, CDKN3, CKAP2, ECT2, NUCKS1, PTTG1, HMGB1-3, HMGN2, and TPX2, suggesting an increased DDR activity. Additionally, the G2M subpopulation was predicted to maintain a low P53 signaling activity (Supplementary Figure 4B), highlighting its distinct properties compared to other NEPCa subpopulations.

The TOX sub-population was distinguished by the high expression of Thymocyte selection-associated HMG box gene (TOX). These cells exhibited heightened activity in the VEGF signaling pathway and expressed elevated levels of TCF4, a transcription factor of Wnt signaling (Supplementary Figures 4B-D).

The STAT3 sub-population not only expressed elevated levels of STAT3 mRNA but also displayed heightened activity in the JAK-STAT signaling, along with increased transactivation of STAT3 target genes (Supplementary Figures 4B-D). Active JAK/STAT signaling is known to promote cellular plasticity[3], suggesting that the STAT3 subpopulation could be driven by JAK-STAT3 signaling.

The NEUROD1 sub-population was characterized by the elevated expression of NEUROD1, GRID2 and GKAP1. It exhibited heightened activities in TGF, EGFR, MAPK, PI3K, ER, and Wnt signaling pathways (Supplementary Figure 4B). The enrichment of Wnt signaling in this subpopulation aligns with a previous study demonstrating that Wnt signaling activates NEUROD1 expression during neurogenesis in mice[4]. In contrast, the expression of ASCL1, a driver transcription factor of NEPCa[5], was detected in all the NEPCa subpopulations except NEUROD1. By examining the feature plots of NEUROD1 and ASCL1 in HuPSA, we observed a mutually exclusive expression pattern between NEUROD1 and ASCL1 in NEPCa (Supplementary Figure 4D).

Supplementary References

1 Song, H. *et al.* Single-cell analysis of human primary prostate cancer reveals the heterogeneity of tumor-associated epithelial cell states. *Nature communications* **13**, 141 (2022).

2 Henry, G. H. *et al.* A cellular anatomy of the normal adult human prostate and prostatic urethra. *Cell reports* **25**, 3530-3542 (2018).

3 Chan, J. M. *et al.* Lineage plasticity in prostate cancer depends on JAK/STAT inflammatory signaling. *Science*, eabn0478 (2022).

4 Kuwabara, T. *et al.* Wnt-mediated activation of NeuroD1 and retro-elements during adult neurogenesis. *Nature neuroscience* **12**, 1097-1105 (2009).

5 Nouruzi, S. *et al.* ASCL1 activates neuronal stem cell-like lineage programming through remodeling of the chromatin landscape in prostate cancer. *Nature Communications* **13**, 2282 (2022).

Supplementary Figure

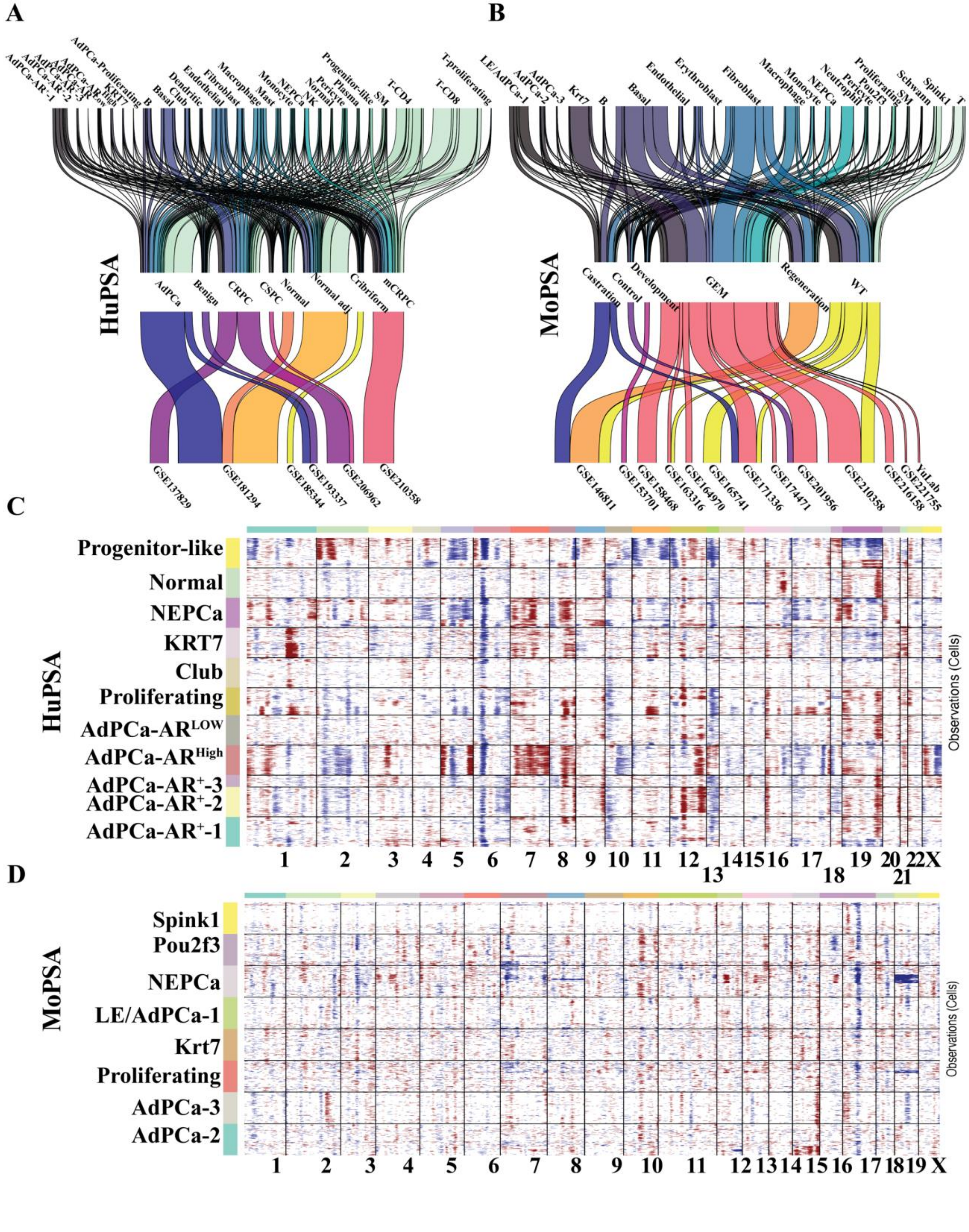

Supplementary Figure 1 Sankey plots of HuPSA (A) and MoPSA (B) to depict the data flow from the newly- defined cell populations (left) to histology groups (middle) and the studies that collected these data (right). InferCNV analysis of the epithelial populations in both HuPSA (C) and MoPSA (D).

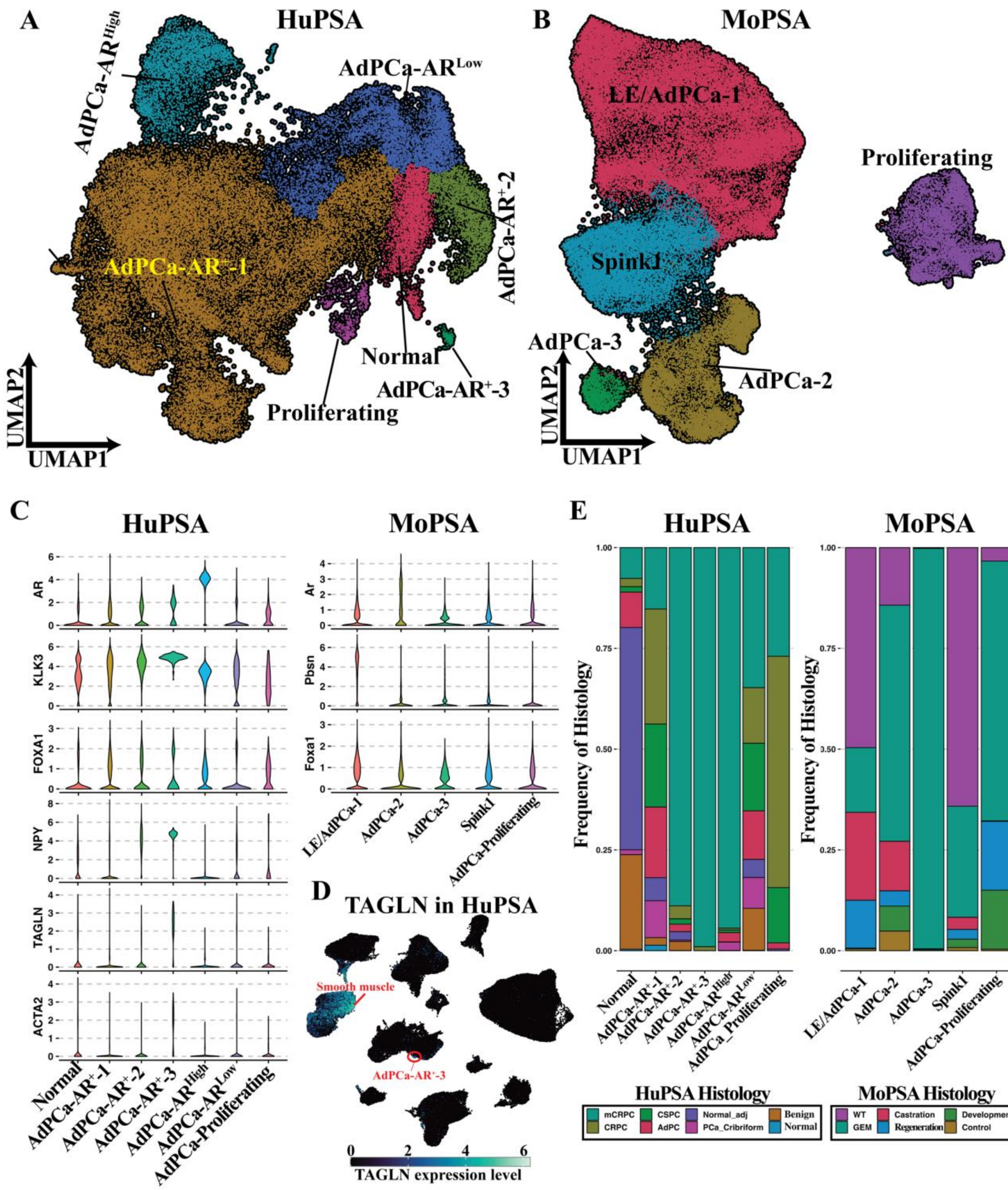
A
HuPSA
AdPCa-AR^{High}
AdPCa-AR^{Low}
AdPCa-AR^{+}-1
AdPCa-AR^{+}-2
Normal
AdPCa-AR^{+}-3
Proliferating
UMAP1
UMAP2
B
MoPSA
LE/AdPCa-1
Proliferating
Spink1
AdPCa-3
AdPCa-2
UMAP1
UMAP2
C
HuPSA
AR
KLK3
FOXA1
NPY
TAGLN
ACTA2
Normal
AdPCa-AR^{+}-1
AdPCa-AR^{+}-2
AdPCa-AR^{+}-3
AdPCa-AR^{High}
AdPCa-AR^{Low}
AdPCa-Proliferating
MoPSA
Ar
Pbsn
Foxa1
LE/AdPCa-1
AdPCa-2
AdPCa-3
Spink1
AdPCa-Proliferating
D
TAGLN in HuPSA
Smooth muscle
AdPCa-AR^{+}-3
0 2 4 6
TAGLN expression level
E
HuPSA
Frequency of Histology
0.00 0.25 0.50 0.75 1.00
Normal
AdPCa-AR^{+}-1
AdPCa-AR^{+}-2
AdPCa-AR^{+}-3
AdPCa-AR^{High}
AdPCa-AR^{Low}
AdPCa_Proliferating
MoPSA
LE/AdPCa-1
AdPCa-2
AdPCa-3
Spink1
AdPCa-Proliferating
HuPSA Histology
mCRPC
CRPC
CSPC
AdPC
Normal_adj
PCa_Cribriform
Benign
Normal
MoPSA Histology
WT
GEM
Castration
Regeneration
Development
Control

Supplementary Figure 2 UMAPs of Luminal/AdPCa populations in HuPSA (A) and MoPSA (B). (C) Violin plots to visualize genes expression in the Luminal/AdPCa populations in HuPSA (left) and MoPSA (right). (D) Feature plot to visualize the expression of the TAGLN gene in HuPSA. TAGLN is expressed in both smooth cells and AdPCa-AR+-3 cells. (E) Bar plots to depict the sample composition of each Luminal/AdPCa populations in HuPSA (left) and MoPSA (right).

Supplementary Data 1 Accession numbers for samples included in HuPSA, MoPSA and ProAtlas.

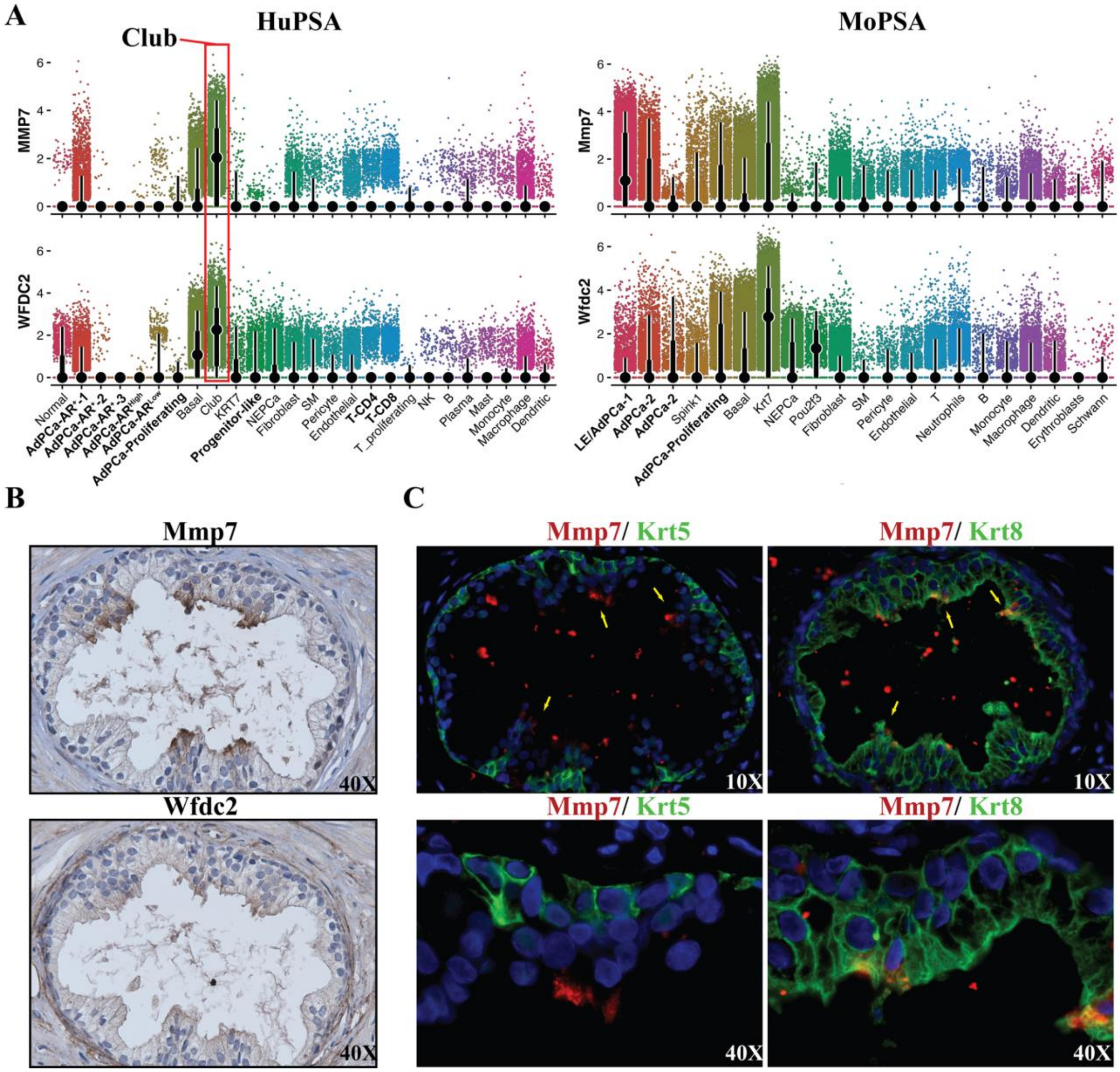

Supplementary Figure 3 Club population. (A) Geyser plots to demonstrate the co-expression of MMP7 and WFDC2 in the Club cells of HuPSA. (B) Immunohistochemistry staining of Mmp7 and Wfdc2 on serial sections confirms the presence of Club cells in human BPH samples. (C) Dual immunofluorescence staining of Mmp7 with luminal epithelial marker Krt8 or basal epithelial marker Krt5 indicates that Mmp7+ cells are luminal but not basal epithelia.

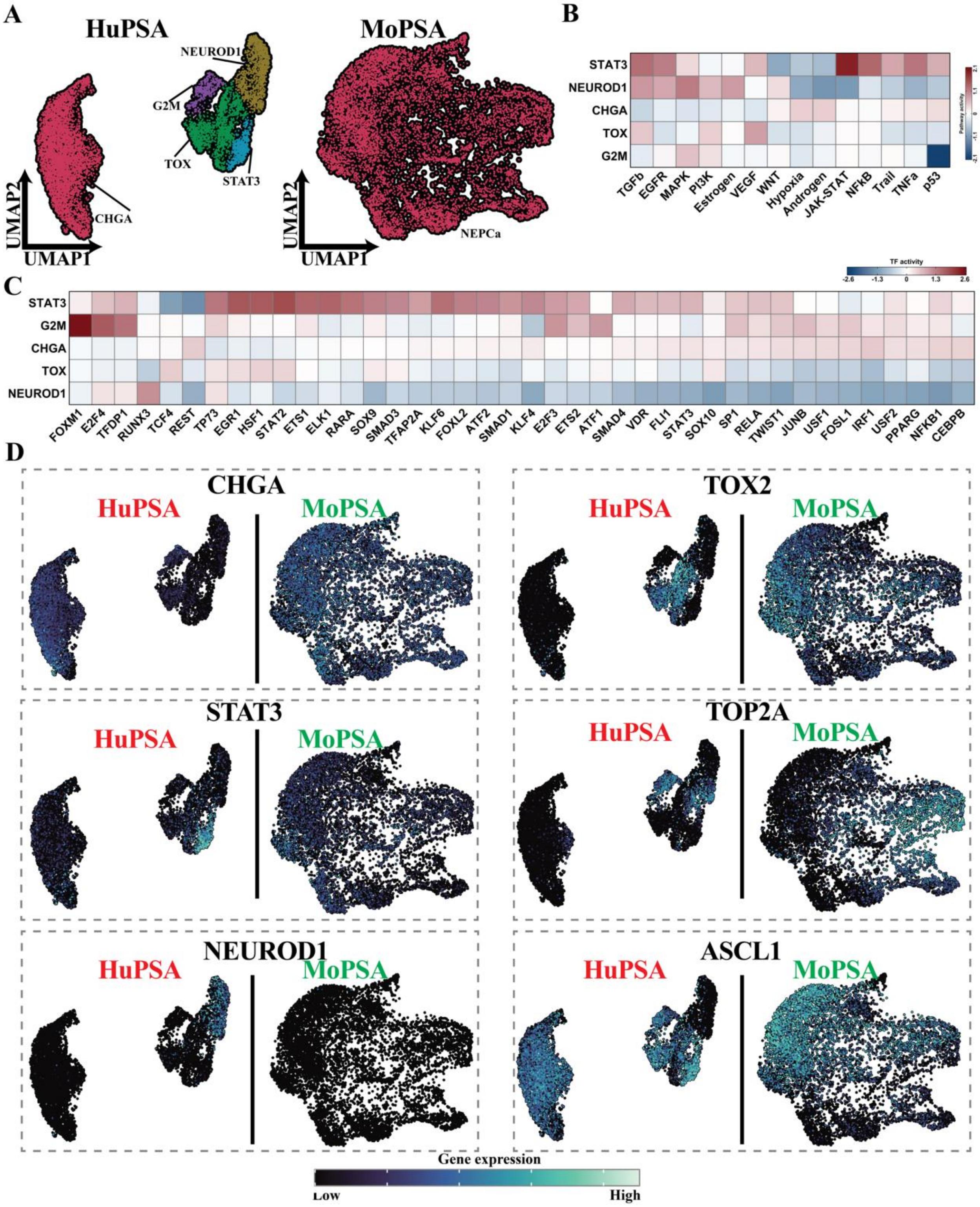
A
HuPSA
NEUROD1
G2M
TOX
STAT3
CHGA
UMAP2
UMAP1
MoPSA
NEPCa
B
STAT3
NEUROD1
CHGA
TOX
G2M
TGFb
EGFR
MAPK
PI3K
Estrogen
VEGF
WNT
Hypoxia
Androgen
JAK-STAT
NFkB
Trail
TNFa
p53
Pathway activity
C
TF activity
-2.6
-1.3
0
1.3
2.6
FOXM1
E2F4
TFDP1
RUNX3
TCF4
REST
TP73
EGR1
HSF1
STAT2
ETS1
ELK1
RARA
SOX9
SMAD3
TFAP2A
KLF6
FOXL2
ATF2
SMAD1
KLF4
E2F3
ETS2
ATF1
SMAD4
VDR
FLI1
STAT3
SOX10
SP1
RELA
TWIST1
JUNB
USF1
FOSL1
IRF1
USF2
PPARG
NFKB1
CEBPB
D
CHGA
TOX2
STAT3
TOP2A
NEUROD1
ASCL1
HuPSA
MoPSA
Gene expression
Low
High

Supplementary Figure 4 NEPCa populations. (A) The UMAP plots revealed heterogenous NEPCa cells in HuPSA and homogenous NEPCa cells in MoPSA. Five NEPCa sub-populations were identified in HuPSA including CHGA, TOX, G2M, STAT3 and NEUROD1. (B & C) Heatmaps illustrate the activities of enriched signaling pathways and upstream transcription factors for the NEPCa sub-populations in HuPSA. (D) Feature plots visualize the expression of marker genes in the NEPCa cells of both HuPSA and MoPSA datasets. It is noteworthy that the expression of ASCL1 and NEUROD1 is exclusive in human NEPCa cells.

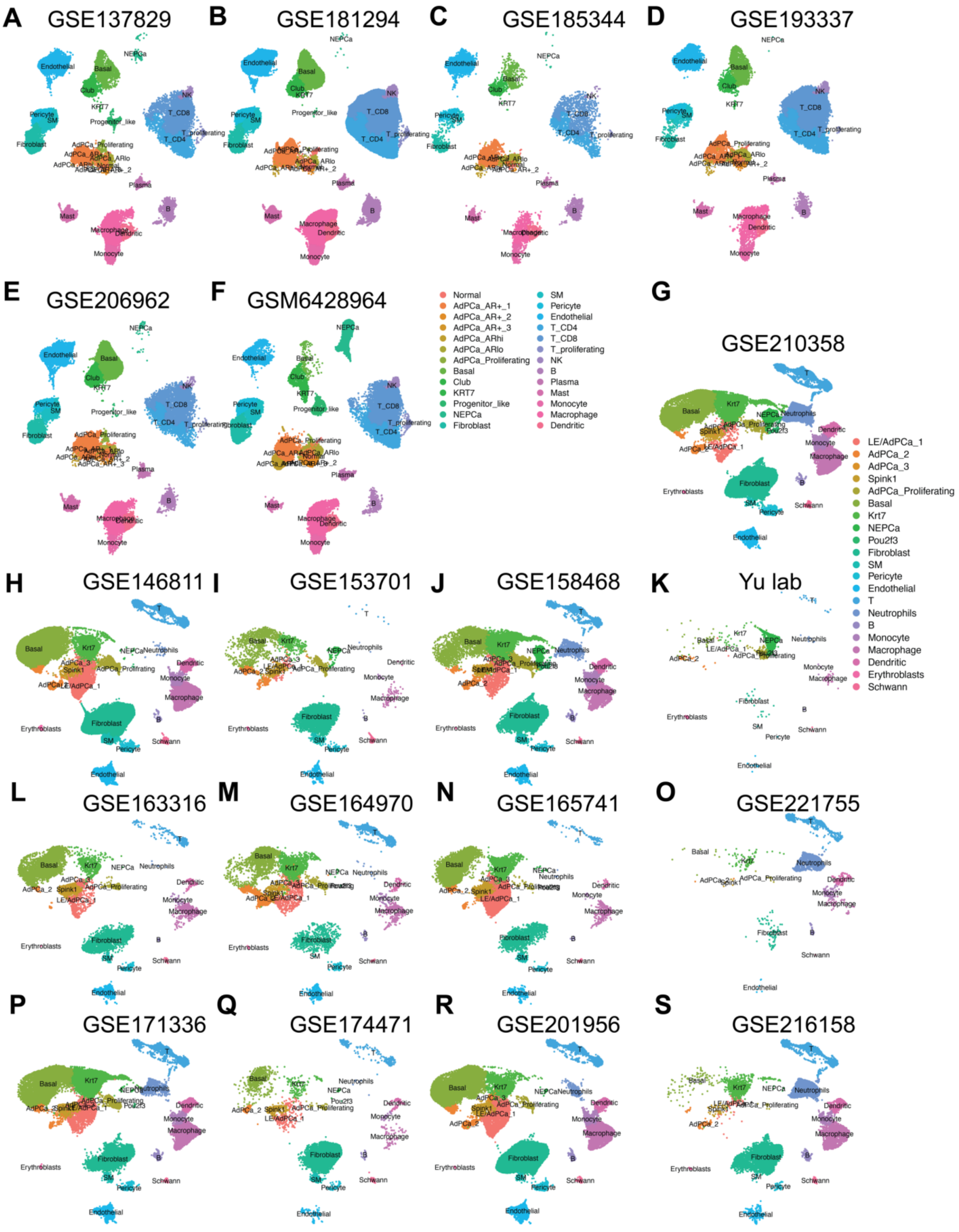

Supplementary Figure 5 Dimensional reduction plots of HuPSA (A-F) and MoPSA (G-S) splited by studies.
